# Psychedelic‐assisted therapy for palliative care within a home treatment setting: A case report

**DOI:** 10.1002/ccr3.9305

**Published:** 2024-08-30

**Authors:** Seragnoli Federico, Martignoni Geo, Martignoni Entela, Silke Bachmann, Rabitti Elisa, Cavuto Silvio, Dubus Zoë, Penzenstadler Louise, Thorens Gabriel, Billieux Joël, Zullino Daniele

**Affiliations:** ^1^ Psychiatry Department Geneva University Hospitals Geneva Switzerland; ^2^ Institute of Psychology University of Lausanne Lausanne Switzerland; ^3^ Private practice Gordola, Ticino Switzerland; ^4^ Department of Psychiatry University Hospitals Halle Saale Germany; ^5^ Psycho‐Oncology Unit Azienda USL‐IRCCS di Reggio Emilia Reggio Emilia Italy; ^6^ Clinical Trial Center‐Statistics Unit, SOC Infrastructure, Research and Statistics Azienda USL‐IRCCS di Reggio Emilia Reggio Emilia Italy; ^7^ Department of History, College of Arts and Science USASK Saskatoon Saskatchewan Canada; ^8^ Medicine Department Geneva University Geneva Switzerland

**Keywords:** home treatment, palliative care, psychedelic, psychedelic‐assisted therapy, throat cancer

## Abstract

**Key Clinical Message:**

This case study describes the feasibility and safety of psychedelic‐assisted therapy (PAT) as a home‐based intervention for a patient with throat cancer experiencing significant existential distress. The patient tolerated the intervention well. This case supports the feasibility and safety of PAT for patients with life‐threatening conditions in a home setting.

**Abstract:**

Psychedelic‐assisted therapy (PAT), as it is practiced today, merges traditional psychotherapeutic techniques with the use of psychedelics such as LSD, psilocybin, or MDMA with the aim of unlocking deeper insights in patients and treating mental conditions that are resistant to other forms of therapy. The present case study describes the safety of PAT as a home‐based intervention for a patient with throat cancer experiencing significant existential distress. The patient tolerated the intervention well and was asked to report on measures of anxiety, depression, and distress related to his somatic condition. The observations provided by this clinical case report align with previous findings, suggesting that PAT can be safely applied to potentially provide relief from existential distress in patients with life‐threatening conditions. As this is a single‐case study, generalizations should be made cautiously. Moreover, placebo effects, expectancy effects, and the natural course of the disease may influence outcomes. Future research should consider controlled trials to ascertain the efficacy and safety of such interventions in diverse settings.

## 1 INTRODUCTION

The re‐emergence of psychedelic‐assisted therapy (PAT) shows promise in the treatment of various psychiatric disorders and existential distress. This article explores a case study of a patient with throat cancer undergoing PAT as a home treatment intervention, detailing the therapeutic process, outcomes, and potential implications for future applications in similar contexts.

### 1.1 Psychedelic substances and their use in therapy

PAT, as it is practiced nowadays, merges traditional psychotherapeutic techniques with the use of psychedelics such as LSD (lysergic acid diethylamide), psilocybin (4‐phosphoryloxy‐NN‐dimethyltryptamine), or MDMA (3,4‐methylenedioxymethamphetamine). The aim is to unlock deeper insights in the patient and treat mental conditions that are resistant to other forms of therapy.^1^, ^2^

So‐called classic psychedelics such as psilocybin and LSD are psychoactive substances that induce a marked modification in the state of consciousness that can be purposefully used in psychotherapy. They are well‐tolerated substances because they have no addictive potential and display a low toxicity profile,^3^ and, unlike opioids or stimulants, they show low potential for physical dependence.^4^ These substances act primarily on serotonergic pathways and do not cause the reinforcing behaviors characteristic of addiction.^5^ Although tolerance may develop with frequent use, requiring increased doses to achieve equivalent effects,^6^ traditional withdrawal symptoms, such as cravings or physical discomfort, are largely absent. Stopping their consumption after prolonged use can lead to transient negative psychological states such as anxiety, but does not carry any other identified risk.^7^

A distinction must be made between classic psychedelics and non‐classic psychedelics such as MDMA. MDMA is considered a “psychedelic” mainly because of its empathogenic, phenomenological effects, which potentially make it a valuable tool for psychotherapy.^8^, ^9^ Nevertheless, its pharmacological action has a different profile in the brain than that of classic psychedelics.^10^ MDMA induces its effects through a potent release of serotonin, dopamine, and norepinephrine.^11^ This release results in heightened emotional empathy, euphoria, and sociability, which are less pronounced with traditional psychedelics. However, the pharmacological action of MDMA also entails significant risks that are more pronounced in recreational settings, including acute hyperthermia and serotonin syndrome, as well as a potential risk of addiction and neurotoxicity with repeated use.^12^, ^13^ Unlike classic psychedelics, which are largely nonaddictive and have a lower physiological risk profile, MDMA's stimulant properties can also lead to increased heart rate and blood pressure, posing additional cardiovascular risks.^14^ Understanding these differential effects and risks is crucial for informed therapeutic use and harm reduction strategies.

As evidenced by a recent systematic review,^15^ the emergence of PAT shows promise in addressing various psychiatric disorders and existential distress. Several recent randomized and controlled clinical trials demonstrate the therapeutic effectiveness of psychedelics in various contexts, in particular in the area of depression,^16^ anxiety,^17^ addiction,^18^ and palliative care.^19^, ^20^

Unlike traditional antidepressant and anxiolytic medications, which take time to have an effect, psychedelics can induce antidepressant and anxiolytic effects within hours of taking them, and they maintain these effects for up to several days after a single administration.^21^

To undergo a PAT session, patients are initially thoroughly screened for suitability. Then, they follow preparatory sessions to establish trust, understand the drug's effects, and determine therapy objectives. The patients' present situation (set) and the context around the substance intake (setting) are also considered.^22^ In addition, the therapist informs the patient about the effects of the psychedelic substance, potential reactions (e.g., changes in sensory perceptions), and strategies to manage them. During the administration phase, psychedelics are given in a controlled, calm setting, with therapists always present and supportive throughout substance administration. Feedback sessions called “integration sessions” are fundamental to anchor the psychedelic‐driven subjective experience to the patient's psychotherapeutic process. This integration process begins immediately after the session and continues over subsequent weeks, helping patients assimilate and apply their new insights to realize and integrate the therapeutic objectives.

### 1.2 PAT situation in Switzerland in 2024: exceptional medical use regimen

Since 2014, the Federal Office of Public Health has issued licenses for the restricted and exceptional medical use of LSD, psilocybin, and MDMA.^23^ In general, a medical application exists if a substance or therapeutic method is used by a physician for a medical indication. Therefore, an application for limited medical use in the context of psychotherapy can be submitted only by a physician and must contain information about the patient, diagnosis, treatment objectives and justification, substance dosage, duration of treatment, and sources of supply. In the current PAT practice in Switzerland,^23^, ^24^, ^25^ participants can receive psilocybin doses ranging from 15 mg to 40 mg, LSD doses ranging from 50 μg to 300 μg, and MDMA doses ranging from 50 mg to 175 mg. The chosen dosage depends on a medical evaluation of the patient's situation. High dosages are considered properly *psychedelic*, as they allow the induction of a potentially intense altered state of consciousness, with the aim of creating a so‐called peak experience. This practice, therefore, is clearly different from microdosing, which is the use of a non‐perceptible dosage of a substance,^26^ or the psycholytic approach, which consists of using a lower dosage of a substance and performing psychotherapy interventions during the alteration of the state of consciousness.^27^ Moreover, clinicians applying for PAT licenses can freely determine the setting and conditions where the therapy takes place, deciding whether it is more suitable for their patients to have a group session^28^ or an individual one.

This application must also be accompanied by the patient's written consent. It is essential to provide the patient with a clear understanding of what to expect in order to reduce anxiety and optimize the chances of having a therapeutically beneficial experience. It is also important to clarify that exceptional medical use of a schedule 1 illicit drug occurs only within the framework of psychotherapy, which is conducted beyond substance intake sessions as part of clinical follow‐up. Finally, it is essential to dispel unreasonable expectations of “miracle drugs” by specifying that, according to available scientific evidence, some patients do not respond favorably to this type of treatment. This treatment is currently reserved for Swiss citizens or people permanently established on Swiss soil (residence permit). Exceptional use authorizations are granted only if (a) the patient has a highly disabling disease, (b) conventional treatments have been exhausted, and (c) the treatment can reignite the therapeutic process and potentially allow the patient to lead a more autonomous lifestyle.

### 1.3 Contraindications and adverse effects in PAT

PAT involves contraindications that must be carefully evaluated before starting the treatment.^29^, ^30^, ^31^ From a somatic perspective, conditions such as severe cardiovascular disease or severe liver cirrhosis are major obstacles to PAT. Specific drug interactions (e.g., those occurring with medications affecting serotonergic receptors such as triptans) can alter the effects of psychedelics. From a psychiatric standpoint, psychotic and bipolar disorders constitute contraindications due to the potential risk of exacerbating symptoms and/or the severity of the disorder. Finally, the lack of a solid support network, in particular the absence of psychotherapeutic care that can help the patient manage potential challenges to their emotional and existential experiences after a PAT session, is also a contraindication to consider. PAT may also involve adverse effects (e.g., transient increase of negative affect or emotional symptoms), which must be mentioned to ensure informed consent is obtained before implementing the treatment.^31^, ^32^

### 1.4 PAT in the context of a life‐threatening diagnosis

Psychological and existential distress is widespread among individuals affected by advanced cancer and other severe life‐threatening or terminal medical conditions. These medical conditions are associated with acute physical and psychological symptoms. Existing treatment modalities for this category of patients, including medication and psychotherapeutic approaches, demonstrate limited effectiveness, especially for psychological and existential distress. When the physical and psychological burden becomes unbearable, people are faced with a condition of global suffering in which, even in the presence of controlled physical pain and adequate emotional relationships, they experience deep anguish whereby the meaning scenarios that have always characterized their life seem insignificant. In such a context, few options remain for the caregiving team, often resulting in the necessity of intermittent sedation or continuous deep palliative sedation. These options effectively reduce consciousness, relieving the patient from the anguish of the dying process. However, for family members and significant others, this situation is extremely challenging and, crucially, limits potential interactions because the patient is sleepy or asleep most of the time.

The World Health Organization has defined existential and spiritual distress domains (such as demoralization syndrome and death anxiety) as fundamental therapeutic targets to enhance the well‐being and quality of life of individuals receiving palliative care.^33^ Demoralization syndrome represents a well‐characterized manifestation of existential distress in response to the stress induced by severe life‐threatening medical conditions. Recognized as a diagnostic entity in the 11th revision of the *International Statistical Classification of Diseases and Related Health Problems* (ICD‐11), it is defined as a mental state of despondency, difficulty in coping, feelings of entrapment, despair, loss of meaning and purpose in life, desire for a hastened death, and functional impairment.^34^ At times, efforts to address existential distress in palliative care through psychosocial, psychopharmacological, and psychotherapeutic interventions yield poor long‐term results. No pharmacological treatments are to date approved for clinical use worldwide that target this type of psychological distress.

### 1.5 Historical perspective of PAT for life‐threatening diagnosis

The idea of administering LSD to make the death of terminally ill patients more “humane”—to reduce anxiety, encourage exchanges with loved ones, and bring about well‐being—did not emerge within the medical profession in the strict sense. The first to write about this possibility was the English writer and philosopher Aldous Huxley. He was a key figure in psychedelic studies: most of the scientists involved in the field at the time referred to his writings. It was during a discussion with him that British psychiatrist Humphry Osmond coined the term “psychedelic” in 1956.^35^ Huxley soon developed the idea that psychedelic experience could be of prime benefit to people at the end of their lives. In 1955, he used a hypnosis technique preceding the death of his first wife, Maria, who died of cancer. Two years earlier, his friend Humphry Osmond had introduced him to mescaline. As early as 1958, in a letter to Osmond, Huxley was very explicit about his project when he stated that “the administration of. LSD to terminal cancer cases, in the hope that it would make dying a more spiritual, less strictly physiological process”,^36^ (p. 15). He elaborated on the idea in his novel *Island*, published in 1962, which quickly became an influential book in both the scientific world and the counterculture of the time.

In 1963, Huxley put his project into practice on himself: suffering from cancer, he asked Laura, his second wife, to administer 100 μg of LSD a few hours before his death,^37^ (p. 283). At his bedside at home were two doctors, including the psychiatrist Sidney Cohen, who had supplied the LSD, and a nurse. All declared that “they had never seen a person in similar physical condition going off so completely without pain and without struggle”.^38^

In the year of Huxley's death, Eric Kast, a specialist in pain assessment and treatment at the Chicago Medical School, hypothesized that LSD, thanks to its ability to disrupt perception, could alter the sensation of pain in his incurable patients.^39^ Kast integrated LSD into treatment for all his patients at the end of life, with more than 300 individuals receiving it in his hospital. In addition to pain relief, Kast observed other favorable effects: some of his patients were less anxious, their moods improved, they communicated better with their loved ones, and they slept more soundly. Following the path of Kast, three other American therapists (Sidney Cohen, Walter Pahnke, and Gary Fisher) experimented with LSD, obtaining similar results and showing positive effects for their patients.^40^ However, these studies do not specify whether some of these patients were cared for at home. This research continued until 1979; notably, the last study of LSD authorized in the USA at the time was for its use in palliative care. Yet, the results of this clinical trial have not been published.^41^

### 1.6 Home treatment

Home treatment has been established for severe psychiatric and somatic disorders. Several advantages of this approach have been determined by research, such as patients' autonomy, benefits from the presence of family and social network, and taking care of social problems in the environment where they arise.^42^ In Geneva, this therapeutic setting was implemented and for the first time applied in the treatment of patients with addictive disorders in 2022. So far, our clinical experience has been encouraging, leading us to transfer our knowledge to PAT in a patient in the terminal stage of malignant disease.

### 1.7 Historical perspective of psychedelic home therapy

Historically, it was not uncommon to implement PAT in patients' own homes. This practice was frequent and accepted during the first wave of Western studies on the topic^43^ and was considered standard by physicians. Apart from clinical trials conducted in medical facilities, many therapists (psychiatrists, psychologists, and psychoanalysts) in the 1950s–1970s used LSD with their private patients, sometimes at their home. One example is American psychologist Betty Eisner,^44^ who was also one of the first psychologists to emphasize that “a bare hospital room is not a good place for a session,” and to recommend that the room where the psychedelic experience takes place be decorated “like the most welcoming home of a friend”.^45^

After the patient had become accustomed to the effects of LSD, some physicians did not hesitate to let them take the substance at home, without medical supervision. American psychiatrist Charles Dahlberg described his treatment of a man with depression. In all, his patient received 26 LSD sessions. From the 21st session onward, Dahlberg decided that it might be beneficial for the patient to take the LSD at home, accompanied only by an attendant, simply to monitor the process. The patient greatly appreciated this change, which also enabled him to work on his relationships with family members.^46^ Sometimes a trusted family member oversaw giving LSD to a patient. English psychiatrist Joyce Martin, who gave LSD to over a thousand patients in 15 years, suggested this to the mother of one of them, pointing out that this woman worked in the medical profession.^47^ American physician Robert Murphy, who had been working with LSD since 1955, reported that he used it “occasionally even unsupervised [at] home, […] without further difficulties of any kind.” He used average doses of 150 μg.^48^

As English psychiatrist Ronald Sandison said in 1959 of one of his patients who was taking LSD, whom he had sent home to be cared for by his mother, “This may not be orthodox, but none of us is doing orthodox things”.^49^ These controversial practices need to be seen in the context of their time, when medical ethics had not yet been established. They do, however, demonstrate how concerned some first‐wave therapists were to their patients' need for comfort and reassurance during the psychedelic experience. Based mostly on their own personal experiences with these substances, they sometimes felt that the home was the most appropriate place for their patients.

The lack of effective interventions to address psychiatric and existential distress today represents a significant limitation in caring for patients with advanced cancer and other severe medical conditions.^15^, ^50^ The present article describes a case study of a patient with throat cancer undergoing PAT as a home treatment intervention. Our objective is to detail the therapeutic process and setting, the clinical outcomes, and the potential implications for future applications in similar contexts.

## 2 CASE HISTORY

A 54‐year‐old man, diagnosed with advanced throat cancer, was treated with LSD. Given the patient's difficulty in motility, it was deemed appropriate to deliver PAT at his residence.

### 2.1 General context

The main responsible psychiatrist had been seeing the patient since April 2022, when he was referred to the psychiatrist by a doctor from the hospice service in the same town. The patient presented with a terminal stage neoplasm that required palliative care at home and regular psychiatric support with visits to his residence every 2 weeks. At the time of our intervention, he was unable to leave his home.

### 2.2 Anamnesis

The patient is the eldest of three children. His parents are divorced, and he lives with his elderly mother. Until the onset of the disease at the beginning of 2020, he led a “normal” life both professionally and personally. He is not married and has no children. He is followed by the oncology unit at the hospital of the nearby town. He currently benefits from a therapeutic support network with home nurses who monitor his health situation daily. In the months prior to treatment, his condition worsened due to the progression of the disease, leading to numerous emergency admissions to the internal medicine department at the nearby hospital. The psychological state of the patient was also severely aggravated with the onset of a major depressive state caused by the somatic condition. The patient was distressed to witness the devastating effects of the tumor with an extremely reduced life expectancy. He agreed to regular specialized care with a full‐dose antidepressant and anxiolytic therapy. Moreover, he was undergoing methadone therapy for pain. The patient was made aware by his physician about the existence and the process of PAT in order to evaluate it as a therapeutic option. In the context of the Swiss exceptional medical use of psychedelics (“compassionate care”), the patient requested undergoing medical treatment with LSD to confront the anxieties of death.

## 3 METHODS

### 3.1 Mental state examination

The patient is a 54‐year‐old male who presented with normovigilance and full orientation across all domains. His speech was coherent, albeit slowed, attributable to an ongoing throat condition. Notably, there were no disturbances in ego perception or thought content. The patient manifested a severely deflated mood and pervasive ruminations on death, which were accompanied by endogenous tensions, sleep disturbances, and significant social withdrawal. There was an absence of dissociative symptoms or psychotic features, and no indicators suggestive of bipolar disorder. The patient reported experiencing pronounced diffuse pain. Despite these considerable challenges, his cognitive functions were largely preserved, he demonstrated a sense of humor, and he exhibited insight regarding his condition.

The patient did not present a pre‐existing psychiatric diagnosis and was never prescribed psychiatric medication before the insurgence of his somatic illness. There was no evidence of a family history for a specific psychiatric disorder.

In his youth, the patient occasionally used psychoactive substances, but never developed an addictive behavior except for tobacco smoking. The patient had smoked tobacco since his twenties.

The patient was not able to leave his apartment, and so no other external stressors were involved in his condition.

### 3.2 Psychiatric diagnosis

The patient was diagnosed as having a severe depressive episode without psychotic symptoms 6A71.3 (ICD‐11). There was no other psychiatric diagnosis.

### 3.3 Somatic diagnosis

The patient was diagnosed with a moderately differentiated squamous cell carcinoma of the posterior region of the tongue, stage IVA T4 N2c M0.

### 3.4 Pharmacological history

From a somatic perspective concerning the terminal illness, the patient received chemoradiotherapy treatment. Given the lack of effectiveness of this treatment, he consequently received an antitumoral intervention with pembrolizumab. This treatment was ongoing at the time of the PAT intervention.

From a somatic perspective concerning pain regulation, the patient received different medications that were ongoing at the time of the PAT intervention:

1. PALLADON INJECT prepared x infusion/injection 20 mg/mL, 16‐0‐0‐0 mg s.c., 16 g/24 h infusion via elastomeric pump for 5 days
2. Ketalgin 5 mg tablet: three tablets (06:00), three tablets (14:00), three tablets (22:00) every 8 h, percutaneous endoscopic gastrostomy (PEG)

From a psychiatric perspective, the patient benefits from psychotherapy provided every 2 weeks at his house. The current psychiatric pharmacotherapy (resumed after discharge from the psychosocial service) is as follows:

1. Escitalopram 20 mg, every day in the morning

### 3.5 Application, dosage, and monitoring during LSD use

This article describes one session of PAT delivered to the patient. In the Swiss context, since one patient receives one authorization to have PAT, it is possible to do multiple sessions. In general, it is deemed necessary to have multiple sessions to increase the durability of the effects of the therapy and the capacity of the patient to integrate change in their daily life. In the Swiss context, the number of sessions proposed generally varies from one to three,^24^ and up to even 12 in the case of therapy that is several years long for patients diagnosed with complex post‐traumatic stress disorder.^25^ There is no standard for how many PAT sessions should be administered because this evaluation is deeply informed by ongoing clinical evaluation on a case‐by‐case basis, which considers improvement and ongoing reaction to the treatment.

The patient was given 100 μg of LSD administered parenterally. The substance was purchased in Switzerland from a certified vendor and stored in a secured refrigerator specially used for this purpose at the main physician office. The patient did not need to halt his other medications. He was constantly monitored during the session, including regular vital sign checks until the psychotropic effect wore off, after about 10 h. In the case of complications (agitation and psychotic symptoms), treatment with lorazepam (Temesta) 2.5 mg p.o. or risperidon (Risperdal) 1 mg p.o. may be administered. If hospitalization is necessary, the patient can be evaluated at the emergency department of the nearby hospital, which is about 10 min from the patient's residence.

### 3.6 Supervision and duration of treatment

During the sessions, in addition to the psychiatrist, a psychologist and a nurse were present. The patient's mother was present in the house during the day of treatment.

The integration session the day after the session was held in the patient's house, in the presence of the physician, nurse, and psychologist. The physician also guaranteed the continuation of psychiatric‐psychotherapeutic care, providing sessions every 2 weeks, which permitted extensive opportunity for the PAT integration process.

### 3.7 Funding

Psychotherapeutic sessions and additional investigations are billed in accordance with the Swiss Health insurance system rate. The treatment is hence reimbursed by insurance, but the substance cost was not covered due to the classification of LSD as an illicit narcotic, not yet recognized as medicine in the pharmacopeia.

### 3.8 Set and setting: qualitative report

The session was conducted by following the Swiss guidelines for PAT^51^ and used a clinical framework developed at the Geneva University Hospitals, which details the theoretical and practical aspects of the different phases of the PAT process.^24^

The session was conducted in the comforting context of the patient's home, specifically in his bedroom with the alternating presence of his mother, a nurse, his psychiatrist, and a psychologist. The setting, a tranquil and familiar environment, was postulated to play a key role in the therapeutic process, allowing the patient to feel safe. The ordinary calm atmosphere and the personal objects around him created a zone of comfort and security for the patient, judged as essential for the therapeutic benefits of the intervention. The patient had tried LSD recreationally a couple of times in his youth at music concerts, and he could still remember some of the consciousness‐altering effects of the substance. When asked about his past experiences, the patient could remember them being positive, while he listened to music with friends. In addition, on further discussion on the matter of music during the preparation session, the patient realized how the effects of these substances could be harnessed here for a different purpose, related to therapy and introspection. We offered the patient the option of listening to music that we could provide or of listening to his own playlist. He decided to choose the music himself and create a playlist of his own taste to experience an existential moment of contemplation about himself and his current health and psychological condition.

During the preparatory session, the patient was able to express his wish and his moderate hope that this experience would provide him with some existential relief. The patient entered the session with a mix of apprehension and hope. Present in the room were always at least two healthcare providers, engaged in ensuring safety and offering psychological support when needed. Their role was to observe and intervene minimally, allowing the patient the space to navigate his own psyche under the influence of LSD. At the beginning of the session, he reported the following things when asked at regular 30‐ to 45‐min intervals: having a positive bodily sensation related to an overall impression of “being fine” and reliving moments of his life as memories came back relative to his friends and partners, coupled with a feeling of nostalgia.

During the session, the patient chose to listen to metal‐rock music, a style he used to play in a band during his adolescence. He had access to a mobile device with downloaded music and no access to the internet. He later described the effect of the music as being amplified in a way he had not felt for years. No complications developed during the session. After 5 h, the patient asked to go to the nearby room to smoke a cigarette, which we agreed to letting him do. During the session, due to his overall physical weakness, the patient was calm and focused on introspection. Any time he opened his eyes, the caregivers actively sought contact and considered a possible need of the patient. The patient responded overall very well to the experience.

In the evening, the caregivers left, leaving the patient to the care of his mother.

## 4 OUTCOME

### 4.1 Integration

The day after the session, during the integration feedback session, the patient thanked the team of caregivers and was able to have a conversation for approximately 35 min before feeling too much fatigue to continue. He was grateful to have been able to get this treatment and to have undergone this experience, saying he preferred to do it at home rather than at the hospital. The physician noted that he was better able to express himself and with more energy than usual during the feedback session, with a better mood. During this conversation, the patient reported having done meditation previously in his life and, although it was a long time ago, he could still use some of the techniques he used to work with to better “entrare lì dentro” (“enter there inside”). He reported an incapacity to put his experience into words and a feeling of amplification of his emotions, which he said was still present a bit during the integration session. He could make jokes during this conversation and reflected on the fact that he would have preferred to have this experience in a group setting rather than by himself. He reported being able during the session to stop thinking about his illness and no longer feeling his body, which was then coupled with a feeling of well‐being. He also, to the surprise of his psychiatrist, reported feelings of ecstasy and peace, which he had not felt for a long time. The patient's mother was also grateful to see her son almost smiling and joking with the healthcare providers, which she had not seen in a long time.

### 4.2 Follow‐up

The patient continued following psychotherapy sessions every 2 weeks after the PAT treatment and did not request any other PAT session. Six months after the PAT session, he appears to be in slowly deteriorating but somewhat stable physical health, as observed in the slow development of the illness and his limited but constant weight loss. Psychologically, despite feeling fatigued, he remains motivated to develop plans that give meaning to his life. He confided that he and his sister are planning a 2‐day trip to the sea in Liguria, a plan that is wholeheartedly supported by the family and the caring team. The patient also feels more empowered to advocate for himself within the medical community and wishes to have a more active role in determining his treatments and care. He is no longer receiving chemotherapy or any other cancer treatment and has expressed a desire to discontinue the use of the morphine release pump. His mother has noted significant improvements over the past few months, and he no longer requires analgesics or anxiolytics. The patient recalls his psychedelic‐assisted treatment positively.

## 5 DISCUSSION

The observations provided by this clinical case report align with previous findings, suggesting that PAT can be safely applied to potentially provide relief from existential distress in patients with life‐threatening conditions.^52^ This result is in line with that of previous research. For example, a pilot study involving psilocybin administration to patients with advanced‐stage cancer showed significant reductions in anxiety and depressive symptoms, highlighting its potential in palliative care.^53^ A randomized controlled trial found that a single dose of psilocybin led to substantial and sustained decreases in anxiety and depression, with improvements in patients' quality of life and emotional well‐being.^17^ In Switzerland, clinical studies that explored LSD‐assisted psychotherapy for anxiety in life‐threatening diseases concluded that LSD was well‐tolerated and significantly reduced anxiety levels at 2 and 12 months post‐treatment.^19^, ^20^ Modern research on the potential of PAT therapy is investigating possible new settings such as PAT group therapy for terminal illnesses,^54^ but such research should always. be accompanied by a consideration of potential challenges and risks.^55^ Adding to this previous research endeavor and specific to this article, we were able to observe that a home treatment setting (i.e., the home environment) was suited to a psychedelic therapy session involving LSD intake.

In particular, given the home treatment setting, a family member was present and able to sometimes assist and support the patient. It is important to recognize that, as an entire psychedelic treatment session lasts as much as 8–10 h, many practical aspects have to be taken care of. For example, accompanying the patient to the toilet, preparing food if needed, being able to provide additional covers for warmth. In this sense, family members who are already taking care of a person daily are well prepared to consider all that is necessary and particularly useful or important for the person in order to create the best comfort for someone they know very well. In fact, the role of peer support (*pair aidant*) provided by family members is more and more recognized as a resource that can be integrated in the clinical process in a fruitful way, both for the patient's health in accompanying and informing the therapists and for the recognition of the role itself and the amount of work provided, which can help family caregivers to stay engaged and resilient in the long run.

Although in the present work the home setting was deemed necessary because of the fragile somatic condition of the patient, we can hypothesize that other indications and motivations could play a role in the decision to provide a psychedelic therapy session at home. These indications may be less related to a somatic impediment to being physically displaced and more focused on psychological issues. Other motives could, for example, be related to the presence of social anxiety or attachment issues. The patient should have the choice to have their psychedelic session administered at home because of their psychological fragility in order to allow family members to support the clinical process. This in turn could present the advantage of the peer supporter being able to better support the patient during the integration process of PAT. Ultimately, lack of financial means could orient a patient to have their psychedelic therapy session at home to avoid part of the cost related to their treatment.

To what extent the setting influences the psychedelic‐induced experience and hence the clinical outcome is yet to be studied systematically. The present work could inform clinical trials that aim to show clinical outcomes based on differences in settings. A crossover design could be imagined with patients receiving the psychedelic treatment session in a standard setting versus a home setting in order to consider potential implications for the clinical outcome. In this framework, a qualitative analysis of the patient's perspectives, observing perceived differences between standard clinical settings and home treatment settings, could inform the definition of the contextual factors, which could be important to consider in improving clinical outcomes.

### 5.1 Limitations

As this is a single‐case study, generalizations should be made cautiously. Moreover, placebo effects, expectancy effects, and the natural course of the disease may influence the outcomes.

If psychometric questionnaires are to be administered, given the unstable physical condition, limited autonomy, and general deterioration of palliative care patients, it is prudent to avoid significant delays in post‐procedure assessments. However, administering some of these measurement tools immediately following a psychedelic experience may have to be carefully evaluated given the psychological vulnerability of the patients at this moment. An effective alternative may be to integrate pre‐ and post‐procedure assessments of performance status depending on the ability of the patients to complete the task. This approach could ensure timely and comprehensive evaluation without overwhelming the patient, if a psychometric evaluation is indeed needed.

### 5.2 Conclusion

This case study underscores the safety of PAT as a home treatment intervention for patients with life‐threatening illnesses. Future research should consider controlled trials to ascertain the efficacy and safety of such interventions in diverse settings.

## AUTHOR CONTRIBUTIONS

**Seragnoli Federico:** Conceptualization; investigation; project administration; writing – original draft; writing – review and editing. **Martignoni Geo:** Investigation; writing – review and editing. **Martignoni Entela:** Investigation; writing – review and editing. **Silke Bachmann:** Writing – review and editing. **Rabitti Elisa:** Methodology; Writing – review and editing. **Cavuto Silvio:** Methodology; writing – review and editing. **Dubus Zoë:** Writing – original draft; writing – review and editing. **Penzenstadler Louise:** Writing – review and editing. **Thorens Gabriel:** Writing – review and editing. **Billieux Joël:** Methodology; supervision; writing – review and editing. **Zullino Daniele:** Supervision; writing – review and editing.

## FUNDING INFORMATION

This research did not receive any specific grant from funding agencies in the public, commercial, or not‐for‐profit sectors.

## CONFLICT OF INTEREST STATEMENT

The authors declare that they have no known competing financial interests or personal relationships that could have appeared to influence the work reported in this paper. By declaring no competing interests, the authors affirm that their research findings and interpretations are presented without any undue influence from personal or financial relationships.

## CONSENT

Written informed consent was obtained from the patient to publish this report in accordance with the journal's patient consent policy.

## Data Availability

Data sharing not applicable to this article as no datasets were generated or analysed during the current study.
